# Examining the Relationship Between State Anxiety, Anxiety Sensitivity, and Peer Defending Using Virtual Reality

**DOI:** 10.3390/bs16020252

**Published:** 2026-02-10

**Authors:** Anna MacGillivray, Julia Byron, Ralph Redden, Laura J. Lambe

**Affiliations:** Department of Psychology, St. Francis Xavier University, Antigonish, NS B2G 2W5, Canada

**Keywords:** bullying, peer defending, bystander intervention, anxiety, anxiety sensitivity

## Abstract

Bullying—a form of deliberate aggressive behaviour where one peer causes harm to another in the context of a power imbalance—is among the top threats facing youth. Witnessing bullying can evoke many feelings, including anxiety, especially for individuals who have been victimized of bullying in the past. Anxiety can shape how individuals navigate social situations, including if and how bystanders intervene in bullying situations. The current study examined how previous bullying victimization, state anxiety, and anxiety sensitivity interact to influence defending behaviours while witnessing social exclusion in a virtual reality (VR) environment. Data were collected from 40 undergraduate participants who completed self-report measures and an in-lab VR task where they had the opportunity to defend against social exclusion. Although bullying victimization was unrelated to state anxiety, results of a moderated mediation model indicated that trait anxiety sensitivity moderated the link between state anxiety and peer defending. For those with low anxiety sensitivity, increased state anxiety after witnessing social exclusion predicted higher rates of defending. However, for individuals with high anxiety sensitivity, the opposite pattern was found, such that state anxiety predicted lower rates of defending. Given these findings, bullying prevention programmes should consider incorporating strategies to address anxiety sensitivity to promote peer defending.

## 1. Introduction

Bullying is a form of aggressive behaviour in which a peer repeatedly causes physical, emotional, or social harm to another person in the context of a power imbalance (World Anti-Bullying Forum, 2021). Bullying victimization is linked to an increased risk of negative health, social, and educational outcomes during childhood and adolescence (Hawker & Boulton, 2000; Children First Canada, 2023), and even extending into adulthood (Kaser et al., 2024). Peer defending—the act of someone witnessing bullying victimization and intervening on behalf of their victimized peer—is considered an effective intervention strategy to end bullying (Hawkins et al., 2001; Lambe et al., 2019; Bezerra et al., 2023). Unfortunately, seminal observation research from 1998 suggests only a small portion (11%) of peers actually defend in the context of school bullying (W. M. Craig & Pepler, 1998). Given that peers are present for 85% of bullying episodes in a school setting (W. M. Craig & Pepler, 1998), many bullying prevention initiatives emphasize bystander intervention (peer defending) as a way to reduce bullying and its psychosocial consequences.

There are mixed findings, however, on the benefits of defending. For example, being defended mitigates low mood for those being victimized (Laninga-Wijnen et al., 2024), but does not decrease their future victimization (Laninga-Wijnen et al., 2025). Similarly, while some prevention programmes highlight the benefits of peer defending (Bezerra et al., 2023), other research indicates that programmes without a bystander focus achieve *greater* reductions in victimization (Gaffney et al., 2021). These inconsistent findings highlight the need for research that can inform the development of more effective bullying prevention strategies by clarifying what factors promote successful intervention. One individual factor that may help us better understanding peer defending is anxiety, a common emotional response when witnessing bullying (Barhight et al., 2013; Lambe & Craig, 2022a). The overarching objective of the current study was to explore how previous bullying victimization, state anxiety, and anxiety sensitivity interact to influence defending behaviours while witnessing social exclusion in a virtual reality task.

Social information processing theory offers a framework for understanding how individuals process and interpret social cues and emotional reactions in situations like bullying (Crick & Dodge, 1994; Lemerise & Arsenio, 2000; Lambe & Craig, 2022a). Witnessing peers being victimized is a complex social, cognitive, and emotional experience for youth (Lambe & Craig, 2022a). According to social information processing theory, bystanders engage in a series of steps that occur rapidly or simultaneously. They must (1) attend to and encode social cues, (2) interpret these cues, which includes their own emotions, (3) determine their goals for the situation, which can be influenced by their emotions, (4) generate possible responses, and (5) enact their chosen response (Crick & Dodge, 1994; Lemerise & Arsenio, 2000). Applied to bullying, bystanders must attend to, encode, and interpret both the bullying victimization situation they are witnessing and their own emotional reactions before deciding how to act.

Social information processing theory proposes that individuals enter a social situation with a database of memories and past experiences that impact how they interpret and respond in a given situation (Crick & Dodge, 1994; Lemerise & Arsenio, 2000). When applied to bullying, bystanders that have experienced bullying victimization themselves may process these situations differently. Considerable research has demonstrated that a history of bullying victimization is positively associated with peer defending (Eijigu & Teketel, 2023; Lambe et al., 2019; Lambe & Craig, 2022b). For example, a systematic review by Lambe et al. (2019) identified 12 studies demonstrating a positive correlation between one’s own bullying victimization and peer defending, and longitudinal research shows that youth with higher levels of bullying victimization also self-report higher levels of defending behaviours over time (Lambe & Craig, 2022b). These findings suggest that individuals with a history of bullying victimization may be more motivated to defend. Other research, however, suggests that bullying victimization is positively associated with passive bystanding (being present during bullying without intervening; Eijigu & Teketel, 2023). This suggests individuals with a history of bullying victimization respond to bullying in different ways, with some defending while others do not. This complex interplay between experiences of victimization and defending behaviours highlights the importance of exploring additional variables that could influence this relationship, such as anxiety.

Anxiety is an emotional response characterized by feelings of tension, worry, and physical changes like increased blood pressure (Griffin, 1990; Carlucci et al., 2023) and is elicited in contexts of perceived danger or uncertainty. Indeed, witnessing bullying can trigger a range of emotional responses, including anger, empathy, and anxiety (Barhight et al., 2013; Lambe & Craig, 2022a), which may shape a bystander’s ability and willingness to intervene. Individuals with their own victimization history may feel especially anxious when witnessing bullying, and if they are unable to regulate their emotional distress, they may be unable to intervene. Research suggests that youth with a history of bullying victimization often exhibit heightened levels of trait anxiety (Reijntjes et al., 2010; Hawker & Boulton, 2000; Children First Canada, 2023), which can heighten their emotional responses during social interactions (Leal et al., 2017; Carlucci et al., 2023). Additionally, previous research has demonstrated how trait anxiety can decrease bystanders’ motivation to defend in bullying situations (Jungert & Perrin, 2019). Given that both prior bullying victimization experiences and anxiety are likely to shape bystander behaviour, it is important to understand how their combined effect may influence peer defending.

Importantly, anxiety has both state and trait components. State anxiety refers to the in-the-moment distress in response to a specific situation, which is unique from individual differences in trait anxiety (Leal et al., 2017; Carlucci et al., 2023). State anxiety arises in the moment and can heighten emotional discomfort. This discomfort could potentially motivate intervention by making the bullying seem urgent and distressing. When witnessing bullying, individuals with a history of bullying victimization may exhibit a stronger emotional reaction due to being reminded of their own distressing experiences. This intensified emotional reaction could influence their subsequent behaviour. For instance, when interpreting social and emotional cues (social information processing step 2), they may perceive the situation as more urgent. Similarly, this heightened awareness may make them more inclined to intervene when determining how to act (social information processing step 3). Recognizing and feeling distressed by bullying is a critical first step in intervention, as this emotional distress may amplify the urgency of the situation and motivate defending behaviour.

State anxiety, however, may not universally motivate defending. In some cases, heightened state anxiety may overwhelm individuals, leading to avoidance rather than action. Individuals also have a baseline level of anxiety sensitivity—a relatively stable trait that reflects a person’s tendency to fear the physical, cognitive, and social consequences of anxiety-related symptoms (Reiss et al., 1986; Stewart, 2024). Unlike state anxiety, which is situational, anxiety sensitivity is a predisposition to react more intensely to feelings of anxiety. Individuals with higher levels of anxiety sensitivity are more prone to experiencing debilitating anxiety when faced with stressful situations, leading to greater behavioural inhibition (Raymond et al., 2023; Zvolensky et al., 2002). In the context of witnessing bullying, individuals high in anxiety sensitivity may gravitate toward avoidance behaviours rather than active intervention. In contrast, individuals with lower anxiety sensitivity may view state anxiety as a manageable and temporary reaction to stress, allowing them to harness this arousal as motivation to help. Therefore, individual differences in anxiety sensitivity may moderate how bystanders respond to their emotional cues. While bullying victimization is positively associated with anxiety sensitivity (Steggerda et al., 2023), no research has examined the moderating role of anxiety sensitivity in the context of bullying and peer defending.

Much of the existing research on peer defending is cross-sectional and reliant on self- or peer-report data, which is subject to social desirability and recall biases. One way to address these limitations is to use an experimental paradigm called Cyberball (Williams & Jarvis, 2006), which has been modified to virtual reality (VR) to study peer defending behaviours (Lambe & Craig, 2022a). In this paradigm, players partake in a ball-tossing game and have the opportunity to defend after witnessing social exclusion, which is one of the most prevalent forms of bullying in Canada (W. Craig et al., 2025), including among emerging adults (Kaser et al., 2024). By using Cyberball in a VR environment, it is possible to examine how bystanders react to victimization scenarios in real time, at multiple time points (e.g., during versus after witnessing victimization), helping to reduce recall and social desirability bias. Prior research (Lambe & Craig, 2022a) has successfully used Cyberball-VR to demonstrate the interplay between state vicarious anger and trait empathy in predicting various peer defending behaviours as they unfold in real time.

The current study used Cyberball-VR (Lambe & Craig, 2022a) to investigate the relationship between bullying victimization, state anxiety, anxiety sensitivity, and defending behaviours among undergraduate students. A recent study demonstrated that nearly half (47.1%) of first-year undergraduates experienced bullying victimization within the past six months (Kaser et al., 2024), highlighting the continued importance of understanding peer defending in this age group. By examining the factors that motivate peer defending in real-time, insights from this study can help us better understand the factors that promote or inhibit defending, thereby helping us understand why some individuals intervene while many others do not. We hypothesized that the association between bullying victimization and defending behaviour would be mediated by increased state anxiety after witnessing social exclusion, and this indirect effect would depend on anxiety sensitivity, such that the mediated pathway would be stronger when anxiety sensitivity is low (i.e., moderated mediation).

## 2. Methods

### 2.1. Participants

Data were collected during the 2024–2025 academic year. Participants consisted of 40 undergraduate students, ages 18–25 years old (*M* = 19.17, *SD* = 1.40), recruited from a university in Eastern Canada. The participants self-identified as predominantly white (92.5%) and the majority self-identified as women (70.0%) (see Table 1). Participants were required to be physically able to use VR equipment (i.e., must be physically able to stand up, hold the controllers, and wear the headset). Participants self-selected to partake in the study through the Psychology Department SONA system, poster advertisements, and word-of-mouth. Compensation included entries in a gift card draw and class credit points for introductory psychology students.

### 2.2. Materials and Measures

#### 2.2.1. Cyberball-VR

Cyberball (Williams & Jarvis, 2006) is a computerized social exclusion paradigm in which the participant engages in a ball-tossing game with other “players”. These other players are computer-generated but believed to be real due to a cover study. The standard Cyberball cover story was used to inform participants that the “study aims to examine mental visualization during an online ball-tossing game with other participants from different universities”. During the game, the participant played three rounds of a simple ball-tossing game. The first and final round are *inclusion* rounds where each player receives the ball an approximately equal number of times (i.e., 13–14/40 tosses). During the middle *exclusion* round, the participant witnesses another player (whom they perceive to be real) be excluded from the game (i.e., the excluded player receives three tosses at the start of the round and then does not receive the ball again). As described in Lambe and Craig (2022a), one player is assigned the “observer” role in each round and is instructed to watch the game closely and consider the players’ actions, emotions, and decision-making strategies, as they are asked to send messages to the other players about what they noticed. The participant is always assigned to be the observer during the exclusion round. After this vicarious exclusion, the participant was instructed to send four messages: one to all players, and three private messages to each of their fellow players individually. These observations are open-ended and were coded to reflect the degree to which defending behaviours are present (see below). After each round of the game, the participant also completes a brief state affect questionnaire (see below). Cyberball-VR was administered using the VIVE Focus 3 virtual reality headset and handheld controllers.

#### 2.2.2. Demographics

Participants were asked to report their age (in years), sex assigned at birth (male, female, intersex, prefer not to say), and gender identity (man, woman, I identify my gender as …, prefer not to say). These variables were used to describe the sample.

#### 2.2.3. Bullying Victimization

A modified version of the Olweus Bullying and Victimization scale (Solberg & Olweus, 2003) was used to assess previous bullying victimization experiences. The modified scale has demonstrated psychometric properties and adaptability for undergraduates (Gaete et al., 2021; Kaser et al., 2024; Vaillancourt et al., 2021). Participants were provided a definition of bullying and responded to 12 items asking about their own victimization experiences during the past six months (e.g., “*I was called mean names, was made fun of, or teased a hurtful way*”). Participants respond on a six-point Likert scale (zero = I have not been bullied in this way in the last 6 months, five = more than once a week). Those who report being bullied more than twice in the past six months are classified as having been victimized and coded to reflect this (zero = no victimization, one = victimized). Dichotomizing bullying victimization scores is common, as this is a relatively low frequency behaviour (Kaser et al., 2024; Volk et al., 2017). The measure showed acceptable reliability (α = 0.70).

#### 2.2.4. State Anxiety

To measure participants’ state anxiety after witnessing social exclusion, a modified version of the Discrete Emotions Questionnaire (Harmon-Jones et al., 2016) was administered after each round of Cyberball-VR. This measure presents the participants with a series of emotion words and asks the participant to rate the extent to which they experienced each on a 7-point Likert scale (1 = not at all, 7 = an extreme amount). For this study, we examined the anxiety subscale which includes the items *anxious*, *nervous*, and *worried*. This scale has been used successfully with early adolescents in a similar Cyberball-VR study to measure vicarious emotions after witnessing social exclusion, where the anxiety subscale demonstrated good reliability (α = 0.82–0.88) (Lambe & Craig, 2022a). Items were averaged to compute a total score for each round, allowing us to examine change in state anxiety after witnessing social exclusion. Higher scores reflect higher levels of state anxiety. Internal consistency was assessed separately for each round of Cyberball-VR (α = 0.74 for Round 1, α = 0.76 for Round 2, and α = 0.89 for Round 3).

#### 2.2.5. Anxiety Sensitivity

To assess participants level of anxiety sensitivity, the Anxiety Sensitivity Index-3 (Taylor et al., 2007) was administered in the pre-game questionnaire. The ASI-3 consists of 18 items measuring anxiety sensitivity (e.g., “*When I tremble in the presence of others, I fear what people might think of me*”) and participants are asked to rate how well each item describes them using a 5-point Likert scale (0 = very little, 4 = very much). The ASI-3 has demonstrated excellent psychometric properties, with strong reliability and validity (Taylor et al., 2007), including among undergraduate students (e.g., total score α = 0.89; Galbraith et al., 2022). An overall anxiety sensitivity score was computed by summing the scores for all 18 items. Higher scores reflect higher levels of anxiety sensitivity. The ASI-3 demonstrated strong internal consistency in the present sample (α = 0.90).

#### 2.2.6. Defending Behaviours

Peer defending was coded from participant observations during Cyberball-VR using different types of defending behaviours established by previous research using this task (Lambe & Craig, 2022a). For the current study, peer defending was operationally defined as responses that included a response acknowledging the exclusion (1 = present, 0 = absent), as it was the most common form of defending behaviour (80.5% of participants had scores > 0), compared to 39.0% for solution-focused defending and 48.8% for comforting. For example, “*I just don’t understand why you didn’t pass the ball to player three*” would be coded as peer defending (acknowledgement of exclusion). Codes are mutually exclusive, such that each phrase can only be coded under one defending behaviour; however, one observation may contain more than one phrase (e.g., *Player 3 was left out, you should include them next time”* would be coded for confronting the exclusion and solution-focused defending, respectively; see Lambe and Craig (2022a) and publicly available codebook for details). Each participant has four observations that were independently coded by two trained raters. Interrater reliability was assessed using Cohen’s kappa, with values of *k* = 0.76, indicating high agreement. Ratings across the four observations (public, player 1, player 2, player 3) were summed into a total score (range 0–4), with higher scores reflecting a higher level of peer defending during the Cyberball-VR game.

#### 2.2.7. Manipulation Check

After playing Cyberball-VR, a manipulation check (Zadro et al., 2004; Lambe & Craig, 2022a) was administered to ensure the participant noticed the social exclusion. Participants were asked to estimate the percentage of total throws that each player received using a 0–100 visual analogue scale.

### 2.3. Procedure

Data was obtained during a one-hour study session. Informed consent was obtained from all participants. First, participants completed baseline (pre-Cyberball) questionnaires (demographics, ASI-3 (Taylor et al., 2007) and the Bullying and Victimization scale (Solberg & Olweus, 2003)).

After the pre-game questionnaire, participants played Cyberball-VR. First, a trained RA explained the VR equipment and assisted in getting them set up with the headset and controllers. The participant completed a brief tutorial to orient themselves to the VR equipment and detailed instructions on how to play the Cyberball-VR game. The participants then played three rounds of Cyberball-VR: one round of inclusion, one round of vicarious exclusion, and another round of inclusion. Each round consisted of 40 tosses between three players and one observer who does not play the round. The vicarious exclusion round is designed so that the participant always takes on the role of the observer. During this round, the participant watches as another player is systematically excluded from the game. Vicarious emotions (Harmon-Jones et al., 2016) and qualitative observations (coded as defending behaviours) were collected at the end of each round of Cyberball-VR.

Following completion of Cyberball-VR, participants completed a manipulation check to ensure they noticed the exclusion. Participants were then debriefed about the true purpose and design of the study and completed a post-debriefing consent. Research ethics clearance was granted by the institutional research ethics committee.

### 2.4. Data Analysis

All analyses were conducted using IBM SPSS Statistics (Version 29). First, descriptive statistics were computed to describe the sample and all study variables. Moderated mediation was tested using PROCESS-macro (model 14; Hayes, 2017) within SPSS. Specifically, we estimated the conditional indirect effect of the independent variable (bullying victimization) on the dependant variable (defending) via the mediator variable (state anxiety) at different values of the moderator (anxiety sensitivity). Moderation is indicated by a significant interaction between state anxiety and anxiety sensitivity on defending. Follow-up simple slopes analyses (Park & Yi, 2023) were used to examine the effect of state anxiety on defending at low, average, and high levels of anxiety sensitivity separately. Age, condition, and baseline state anxiety^1^ were entered as covariates, consistent with previous research (Lambe & Craig, 2022a). Unstandardized regression coefficients for all paths are reported. *R*^2^ values and indirect effects are reported as indices of effect sizes.

## 3. Results

### 3.1. Manipulation Check

Participants estimated that Player 3 received the ball significantly less (*M* = 4.00, *SD* = 5.01; *p* < 0.001) than both player 1 (*M* = 51.81, *SD* = 22.66) and player 2 (*M* = 51.79, *SD* = 22.61), who did not differ, *p* = 0.79.

### 3.2. Descriptive Statistics and Correlations

Means, standard deviations, and bivariate correlations are presented in Table 2. Gender and defending were significantly positively correlated, *p* < 0.05, such that women were more likely to demonstrate this behaviour during the task relative to men. While no other bivariate correlations were statistically significant, there was a trend, such that bullying victimization was positively related to defending, *r* = 0.26, *p* = 0.10.

### 3.3. Moderated Mediation Analysis

**Figure 1:**
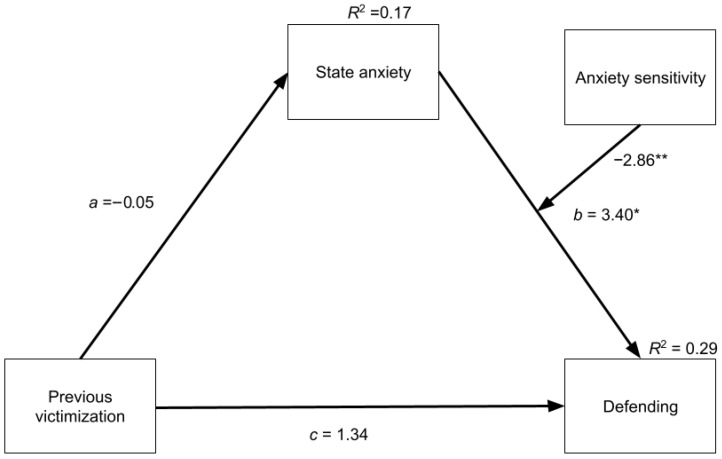
The relationship between previous victimization, anxiety, and defending. *Note.* Moderated mediation model examining the indirect effect of victimization on defending behaviour through state anxiety, with anxiety sensitivity as a moderator of the relationship between state anxiety and peer defending. * *p* < 0.05; ** *p* < 0.01.

A moderated mediation model was conducted to examine the relationships between previous bullying victimization, state anxiety, anxiety sensitivity, and defending behaviours when witnessing social exclusion. As shown in Table 3 and Figure 1, the link between previous victimization and state anxiety (*a* path) was not significant, and the direct effect of victimization on defending behaviours (*c* path) was also not significant. When looking at the *b* path, however, there was a significant positive association between state anxiety and defending, *p* < 0.05. Additionally, there was a positive association between anxiety sensitivity and defending, *p* < 0.05. As hypothesized, there was a significant interaction between anxiety sensitivity and state anxiety on defending, *p* < 0.05. The overall index of moderated mediation was not significant, *b* = 0.14, 95% CI [−1.76, 2.75] *SE* = 1.10, indicating that the indirect effect of bullying victimization on defending through state anxiety did not significantly vary across levels of anxiety sensitivity. Overall, the model accounted for 17% of the variance in state anxiety and 29% of the variance in defending.

### 3.4. Post Hoc Moderation Analysis

**Figure 2:**
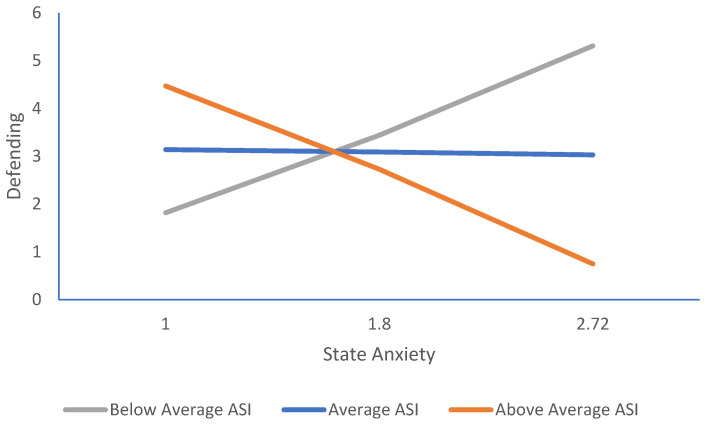
The effect of state anxiety on defending moderated by anxiety sensitivity. *Note.* The effect of state anxiety on defending at low (−1 *SD*), mean, and high (+1 *SD*) levels of anxiety sensitivity.

A post hoc moderation analysis was conducted to simplify the original hypothesized model, given the null effects for bullying victimization. Specifically, we examined whether anxiety sensitivity moderated the link between state anxiety and defending. This model was examined using PROCESS model 1 with age, condition, and Round 1 state anxiety as covariates. As shown in Table 4 and Figure 2, the positive link between both state anxiety (*b* = 3.51, *p* = 0.02) and anxiety sensitivity (*b* = 4.81, *p* = 0.01) on defending was significant. Anxiety sensitivity, however, significantly moderated this effect, *p* < 0.01. Simple slopes analyses were used to probe the significant interaction at one standard deviation above and below the mean values of anxiety sensitivity. When anxiety sensitivity was below average (ASI = 0.53), state anxiety predicted higher levels of defending, *b* = 1.94, *p* = 0.05. In contrast, when anxiety sensitivity was above average (ASI = 2.01), the effect was negative, *b* = −2.43, *p* = 0.01, such that state anxiety predicted lower levels of defending. At the average level of anxiety sensitivity (ASI = 1.17), the effect of state anxiety on defending was not significant. Overall, the model accounted for 24% of the variance in defending.

## 4. Discussion

The purpose of this study was to examine how previous bullying victimization, state anxiety, and anxiety sensitivity interact to influence defending behaviours while witnessing social exclusion. While the overall moderated mediation model was not significant, a key finding of the study is the role of anxiety sensitivity as a moderator between state anxiety and defending behaviours. Specifically, the direction of the link between state anxiety and defending depended on levels of anxiety sensitivity. This suggests that while state anxiety may prompt some individuals to intervene, those with high anxiety sensitivity may be less likely to act in threatening situations like bullying. Although the full model did not support the hypothesized indirect effect, the significant moderation aligns with our prediction regarding anxiety sensitivity, offering a novel contribution: reducing anxiety sensitivity may help individuals engage more in defending behaviours when witnessing bullying or exclusion.

Anxiety sensitivity can play a crucial role in how individuals process and respond to social situations. Those with high anxiety sensitivity tend to perceive their own symptoms of anxiety as threatening, which may lead them to avoid situations that could heighten their distress. In contrast, individuals with low anxiety sensitivity may experience anxiety as a motivator, pushing them to take action. These findings align with research suggesting that high anxiety sensitivity is linked to avoidance behaviours (e.g., Lebowitz et al., 2015; Lehrbach et al., 2023), and influences behavioural responses to perceived threats (e.g., Raymond et al., 2023). Our findings also align with social information processing theory (Crick & Dodge, 1994; Lemerise & Arsenio, 2000; Lambe & Craig, 2022a) by suggesting that emotional factors, such as anxiety sensitivity and state anxiety, influence how individuals process and react (both cognitively and/or emotionally) to social cues, which impacts their actions. In the context of bullying, individuals who have a stronger fear of anxiety sensations may be more likely to freeze and remain passive, or flee the situation altogether, rather than confront the situation. These results underscore the importance of considering individual differences when developing anti-bullying strategies that rely on bystander intervention. Our results highlight the need to rethink blanket approaches that encourage all students to intervene, as not all students have the coping skills necessary to defend when witnessing bullying. Research by Healy (2020) suggests that universal bystander intervention may produce iatrogenic results, inadvertently causing harm to both those being victimized and the bystanders who feel ill-equipped to defend. To address this, anti-bullying programmes could focus on more targeted encouragement to intervene, possibly based on social dynamics or relationships. Alternatively, schools could incorporate anxiety management training into existing anti-bullying programmes or as a part of regular mental health education or social emotional learning initiatives. As anxiety sensitivity is a modifiable trait (Stewart, 2024), providing individuals with skills to cope with their anxiety sensations when witnessing bullying may have implications for increasing bystander intervention rates.

Our results did not provide support for the hypothesis that previous victimization experiences predict defending behaviour after witnessing bullying. While many previous studies with adolescents have found a link between victimization and defending (Eijigu & Teketel, 2023; Lambe et al., 2019; Lambe & Craig, 2022b), other studies have not found this association (e.g., van der Ploeg et al., 2017), suggesting their connection is not necessarily straightforward. One possible explanation for this result is that bullying tends to peak during early adolescence and decline over high school years (Vaillancourt et al., 2023), meaning the frequency and salience of past bullying experiences may be lower in our emerging adult sample. This low prevalence, combined with our relatively small sample size, may have impacted our ability to detect an effect. Furthermore, this study only examined bullying victimization experiences within the past six months. Future research could consider examining a broader scope of victimization experiences to further examine the relationship between this factor and defending. Additionally, while negative feelings such as anxiety did arise for some participants after witnessing victimization, this was not specific to individuals who reported a bullying victimization. This finding may be explained by the idea that anxiety is already prevalent in this population (e.g., 64.5%; American College Health Association, 2016). If anxiety levels are already elevated, additional anxiety triggered by past experiences of victimization may not result in a significant increase.

While research is beginning to explore the use of VR as a tool for understanding bystander behaviour (e.g., see review by Xue et al., 2021), this is the first study to examine state anxiety’s impact on defending behaviours after witnessing a simulated bullying scenario in the lab. Prior bullying research work has primarily relied on retrospective, self-report measures, and hypothetical scenarios (e.g., see review by Lambe et al., 2019), with newer research beginning to incorporate VR technology (e.g., Lambe & Craig, 2022a; Liu et al., 2023, 2025). By using Cyberball-VR, we were able to measure participant’s in-the-moment distress immediately after witnessing social exclusion. Unlike retrospective self-report measures, which rely on memory, this approach captures real-time emotional reactions. This offers a clearer picture of how individuals feel after witnessing bullying, and how their emotions impact their subsequent behaviours.

### Limitations and Future Research

One key limitation of this study is the relatively small and homogeneous sample of university students. Our analyses were likely underpowered to detect small-to-moderate indirect effects. Future studies should aim for a larger, more diverse sample to increase the generalizability of the findings. Future research should also explore the effects of bullying victimization and anxiety on defending behaviours across different populations, such as adolescents, who are more sensitive to peer stressors and whose experiences of bullying victimization may be more recent/salient (Mullan et al., 2023). While Cyberball-VR effectively elicited defending behaviours and changes in state anxiety, there are still several limitations of this paradigm. First, participants in the study interact with unknown players with whom they have no prior relationship or history. Although our results provide valuable insights into the predictors of peer defending, our paradigm does not consider relationships or aspects of the broader school atmosphere, which are related to peer defending (Konishi et al., 2021; Lambe & Craig, 2022b). The social exclusion in Cyberball-VR is also relatively benign. The distress experienced by peer bystanders when witnessing bullying in real life is likely to be much greater than can be reproduced in the lab. Additionally, the coding of verbal observations as defending behaviours does not capture the tone of voice or non-verbal cues during defending behaviours, both of which can alter the meaning of an interaction. Future studies could incorporate methods such as video recordings, prosodic analysis of audio, or trained observers to capture these nuances for a more comprehensive understanding of defending behaviours.

Furthermore, while this study focused on anxiety, previous research has demonstrated that emotions like anger can motivate peers to defend (Lambe & Craig, 2022a). Bullying victimization often leads to individuals internalizing their experiences and feeling emotions such as shame or fear (Mullan et al., 2023). Interventions such as Emotion-Focused Therapy (Greenberg, 2004; Marren et al., 2022) could help replace these feelings with more adaptive emotions, such as anger about their victimization, which may increase the likelihood of peer defending or other approach-oriented coping strategies. Future research should explore whether targeting the emotions of bystanders, particularly those who have experienced bullying victimization in the past, can enhance peer defending.

## 5. Conclusions

This study examined how bullying victimization, state anxiety, and anxiety sensitivity interact to influence defending behaviours while witnessing social exclusion. While our findings did not support the hypothesized moderated mediation model, findings do suggest that the relationship between state anxiety and defending is moderated by anxiety sensitivity. In other words, our findings demonstrate that state anxiety can have a differential effect on peer defending depending on the bystander’s level of anxiety sensitivity. For those with low anxiety sensitivity, state anxiety can facilitate defending. However, individuals with high anxiety sensitivity may struggle to intervene in threatening situations like bullying.

By using a novel in-lab design, this study was able to capture the nuanced effects of anxiety on peer defending behaviour in ways not observable through self- or peer-report data alone. This approach provides new insight into how momentary emotional states can influence bystander’s decisions, and how individual differences in anxiety sensitivity can shape these responses. Understanding these processes not only advances the literature but can also have practical implications for bullying interventions that rely on bystanders. By positioning anxiety sensitivity as both a modifiable mental health risk factor and barrier to bystander intervention, addressing this trait among youth could foster adaptive mental health skills and a more supportive school environment where peer defending occurs.

## Figures and Tables

**Table 1:** Demographics.

	N	%	Mean	SD
Age	40		19.17	1.40
*Gender*				
Women	28	70.0		
Men	12	30.0		
*Ethnicity*				
White	37	92.5		
Asian	3	7.5		

**Table 2:** Descriptive statistics and correlations.

	Victimization	Δ State Anxiety	Peer Defending	Anxiety Sensitivity	Age	Gender
Victimization	--					
Δ State anxiety	0.01	--				
Peer defending	0.26	−0.14	--			
Anxiety sensitivity	−0.01	−0.05	0.23	--		
Age	−0.23	0.06	−0.08	−0.17	--	
Gender	0.20	0.01	0.34 *	0.10	−0.20	--
Mean/Frequency	63% victimized	0.32	2.83	1.22	19.17	65.2% women 34.8% men
SD		1.01	0.49	0.74	1.40	

*Note*. Δ State anxiety is a change score (Round 2–Round 1). Gender was coded as men = 1, women = 2. * *p* < 0.05.

**Table 3:** Moderated mediation analysis.

	M (State Anxiety) *b* [95% CI]	SE	t	*p*	Y (Defending) *b* [95% CI]	SE	t	*p*
*Predictors*								
Intercept	0.78 [−3.87, 5.43]	2.29	0.34	0.74	−1.27 [−13.63, 11.09]	6.07	−0.21	0.84
Victimization (*a* path)	−0.05 [−0.68, 0.58]	0.31	−0.16	0.87	1.33 [−0.34, 3.00]	0.82	1.62	0.12
State anxiety (*b* path)					3.40 * [0.43, 6.36]	1.46	2.33	0.03
Anxiety sensitivity					4.83 *** [1.63, 8.04]	1.57	3.07	0.00
Interaction State anxiety × anxiety sensitivity					−2.86 ** [−5.02, −0.69]	1.06	−2.68	0.01
*Covariates*								
State anxiety (Round 1)	0.41 [0.02, 0.80]	0.19	2.13	0.04 *	0.71 [−0.45, 1.87]	0.57	1.25	0.22
Condition	0.34 [−0.03, 0.70]	0.18	1.89	0.07	−0.27 [−1.28, 0.73]	0.50	−0.55	0.58
Age	−0.01 [−0.24, 0.21]	0.11	−0.12	0.90	−0.14 [−0.76, 0.47]	0.30	−0.48	0.64
Model *R*^2^	0.17				0.29			
*F* (*df*)	1.83 (4, 35)				1.91 (7, 32)			

*Note*. Unstandardized coefficients are presented. N = 40. * *p* < 0.05; ** *p* < 0.01; *** *p* < 0.001.

**Table 4:** Moderation Analysis.

	Defending *b* [95% CI]	SE	t	*p*
Predictors				
Intercept	1.87 [−10.12, 13.86]	5.89	0.32	0.75
State anxiety	3.51 * [0.48, 6.54]	1.49	2.35	0.02
Anxiety Sensitivity	4.81 ** [1.53, 8.09]	1.61	2.99	0.01
Interaction State anxiety × anxiety sensitivity	−2.95 ** [−5.16, −0.74]	1.09	−2.71	0.01
*Covariates*				
State anxiety (Round 1)	0.86 [−0.31, 2.03]	0.58	1.50	0.14
Condition	−0.15 [−1.16, 0.87]	0.50	−0.29	0.87
Age	−0.29 [−0.89, 0.32]	0.30	−0.96	0.34
Model *R*^2^	0.24			
*F* (*df*)	1.71 (6, 33)			

*Note*. Unstandardized coefficients are presented. N = 40. * *p* < 0.05; ** *p* < 0.01.

## Data Availability

Data and syntax are publicly available on Open Science Framework https://osf.io/wxspe/overview?view_only=251daa27e5a143109be179ded5b7502f.

## Note

1 Age was entered as a covariate as it is negatively correlated with defending. Round 1 state anxiety was entered to control for baseline state anxiety. Condition was entered as a covariate because this study was part of a larger project examining group dynamics, in which participants were assigned to either an in-group victimized condition, an in-group aggressor condition, or a control condition. As shown in Tables 3 and 4, there were no significant condition effects.
